# Author Correction: PRL3-zumab as an immunotherapy to inhibit tumors expressing PRL3 oncoprotein

**DOI:** 10.1038/s41467-021-26548-6

**Published:** 2021-11-02

**Authors:** Min Thura, Abdul Qader Al-Aidaroos, Abhishek Gupta, Cheng Ean Chee, Soo Chin Lee, Kam Man Hui, Jie Li, Yeoh Khay Guan, Wei Peng Yong, Jimmy So, Wee Joo Chng, Chin Hin Ng, Jianbiao Zhou, Ling Zhi Wang, John Shyi Peng Yuen, Henry Sun Sien Ho, Sim Mei Yi, Edmund Chiong, Su Pin Choo, Joanne Ngeow, Matthew Chau Hsien Ng, Clarinda Chua, Eugene Shen Ann Yeo, Iain Bee Huat Tan, Joel Xuan En Sng, Nicholas Yan Zhi Tan, Jean Paul Thiery, Boon Cher Goh, Qi Zeng

**Affiliations:** 1 Institute of Molecular and Cell Biology, Agency for Science, Technology and Research (A*STAR), Singapore, 138673 Singapore; 2 Department of Haematology-Oncology, National University Cancer Institute, Singapore (NCIS), Singapore, 119082 Singapore; 3 Division of Cellular and Molecular Research, National Cancer Centre Singapore, Singapore, 169610 Singapore; 4 Department of Medicine, Yong Loo Lin School of Medicine, National University of Singapore, Singapore, 119260 Singapore; 5 Division of Surgical Oncology, National University Cancer Institute, Singapore (NCIS), Singapore, 119082 Singapore; 6 Cancer Science Institute of Singapore, National University of Singapore, Singapore, 117599 Singapore; 7 Department of Urology, Singapore General Hospital, Singapore, 169608 Singapore; 8 Division of Medical Oncology, National Cancer Centre Singapore, Singapore, 169610 Singapore; 9 Lee Kong Chian School of Medicine, Nanyang Technological University, Singapore, 308232 Singapore; 10 Department of Colorectal Surgery, Singapore General Hospital, Singapore, 169608 Singapore; 11 Department of Biochemistry, Yong Loo Lin School of Medicine, National University of Singapore, Singapore, 119260 Singapore

**Keywords:** Cancer immunotherapy, Drug development, Antibodies, Tumour immunology

Correction to: *Nature Communications* 10.1038/s41467-019-10127-x, published online 06 June 2019.

In this article the funding from ‘Open Fund-Individual Research Grant (OF-IRG), National Medical Research Council (NMRC) Singapore with the project ID of NMRC/OFIRG/0053/2017’ was omitted.

