# Supplementary material for: PRL3-zumab as an immunotherapy to inhibit tumors expressing PRL3 oncoprotein
Source: Nat Commun. 2019 Jun 6;10:2484. doi: 10.1038/s41467-019-10127-x (PMC6554295; doi:10.1038/s41467-019-10127-x)
Supplement: Supplementary file 1 — Supplementary Information [file 41467_2019_10127_MOESM1_ESM.pdf]

Supplementary Information

PRL3-zumab as a unique immunotherapy to inhibit tumors expressing PRL3 oncoprotein

Thura et al

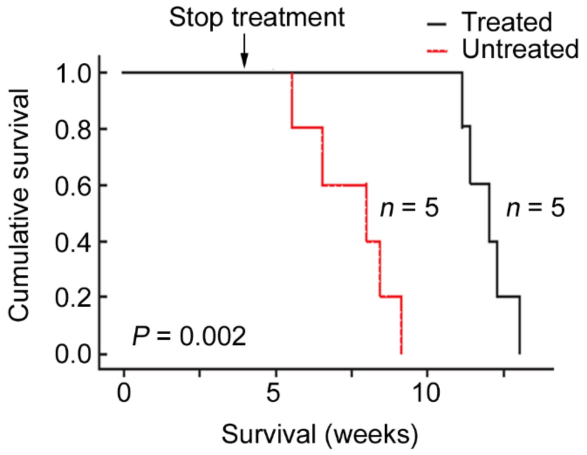

Supplementary Figure 1. PRL3-zumab prolongs survival of mice harboring PRL3$^+$ MHCC-LM3 orthotopic liver tumors. Kaplan Meier survival analysis of untreated (red lines) and treated (black lines) groups of mice. *n* = 5 per group; *P* = 0.002, log-rank test. Source data are provided as a Source Data file.

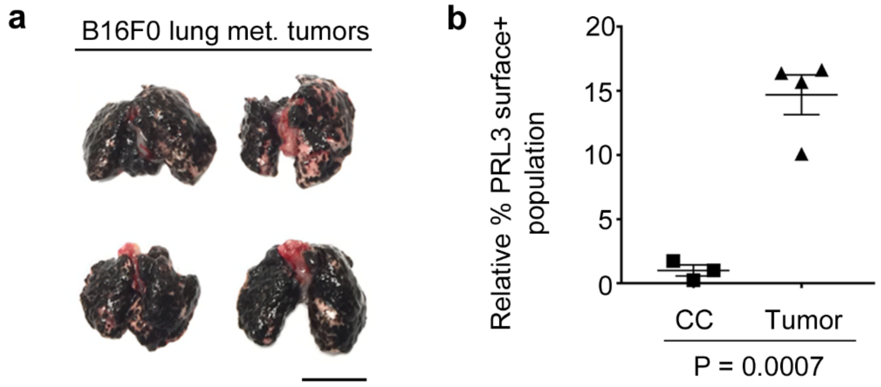

Supplementary Figure 2. The surface+ PRL3 cell population is more abundant in B16F0 tumors *in vivo* compared to B16F0 cultured cells *in vitro*. (**a**) Images of metastatic lung tumors formed by tail-vein injection of $PRL3^{+}$ B16F0 melanoma cells. Scale bar, 10 mm. (**b**) Flow cytometry analysis reveals higher cell-surface PRL3 expression in B16F0 metastatic lung tumors (n = 4 independent samples) compared to cultured cells (CC; n = 3 independent samples). Tumor volumes were analyzed using the *t* test (mean ± s.e.m.), P = 0.0007. Source data are provided as a Source Data file.

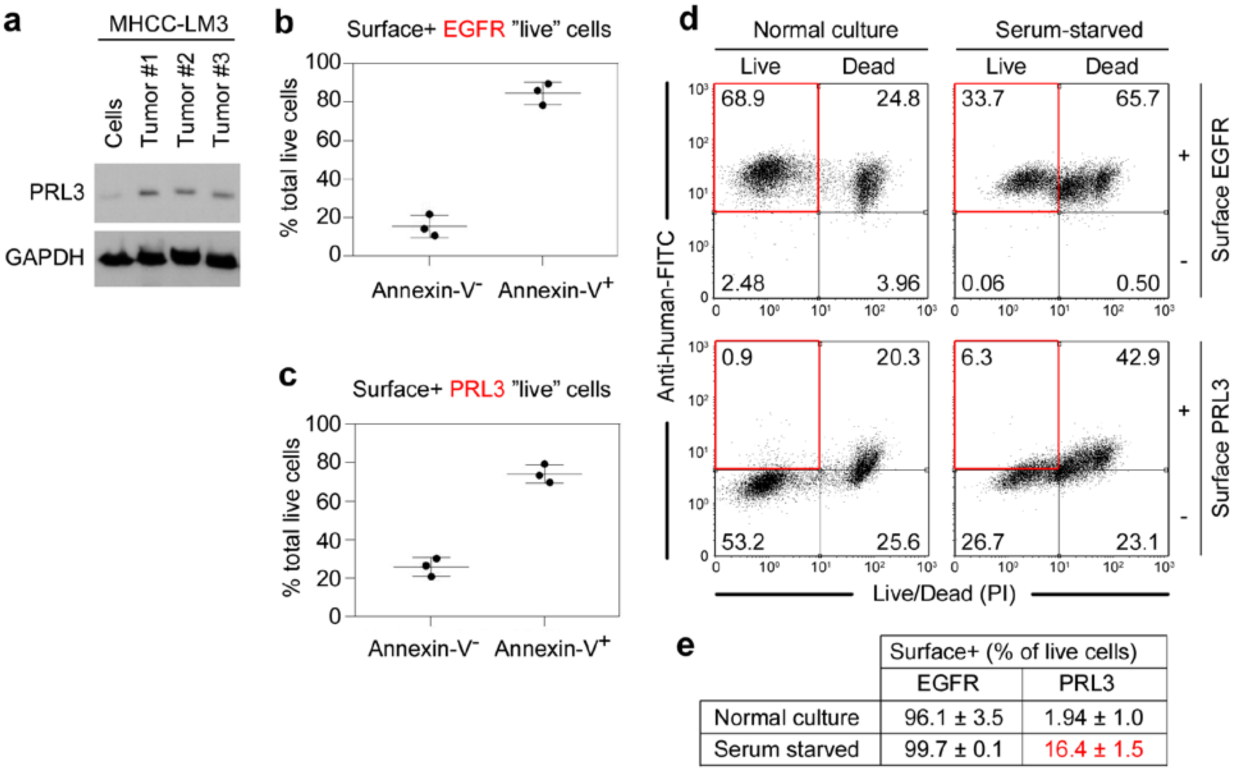

	Surface+ (% of live cells)	
	EGFR	PRL3
Normal culture	96.1 ± 3.5	1.94 ± 1.0
Serum starved	99.7 ± 0.1	16.4 ± 1.5

Supplementary Figure 3. Surface PRL3 is expressed on viable and early apoptotic tumor cells *in vitro* and increased upon serum starvation. (**a**) PRL3 immunoblot of total lysates from MHCC cultured cells and three independent tumors. GAPDH served as a loading control. (**b, c**) Independent surface+ tumor cell populations for EGFR (**b;** *n* = 3) or PRL3 (**c;** *n* = 3) were analysed for the proportion of viable (Annexin-V$^-$) and early apoptotic (Annexin-V$^+$) cells. Data presented as mean ± s.d.. (**d**) MHCC cells cultured under normal culture or serum starvation (72 h) were analysed for surface EGFR and PRL3 expression. The mean percentage ± s.d. of surface-positive live cells out of the total live cell population is shown in the top left quadrant of each representative FACS profile. (**e**) Mean percentage ± s.d. of surface positive (surface+) live cells in (**d**) were calculated by dividing the surface antigen-positive live cells (upper left quadrant) by total live cells (sum of both upper and lower left quadrants). Source data are provided as a Source Data file.

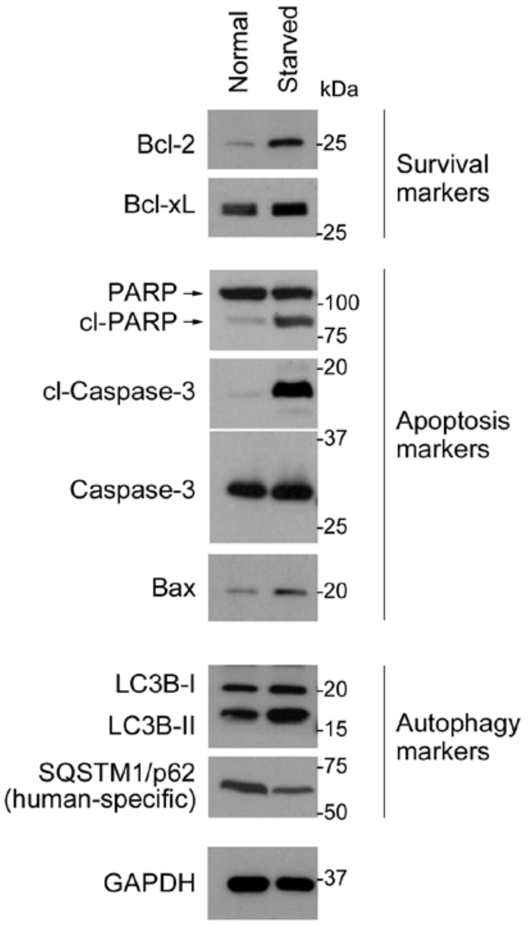

Supplementary Figure 4. Serum-starvation induces the activation of pro-survival, pro-apoptotic and autophagy pathways in MHCC-LM3 cells. Source data are provided as a Source Data file.

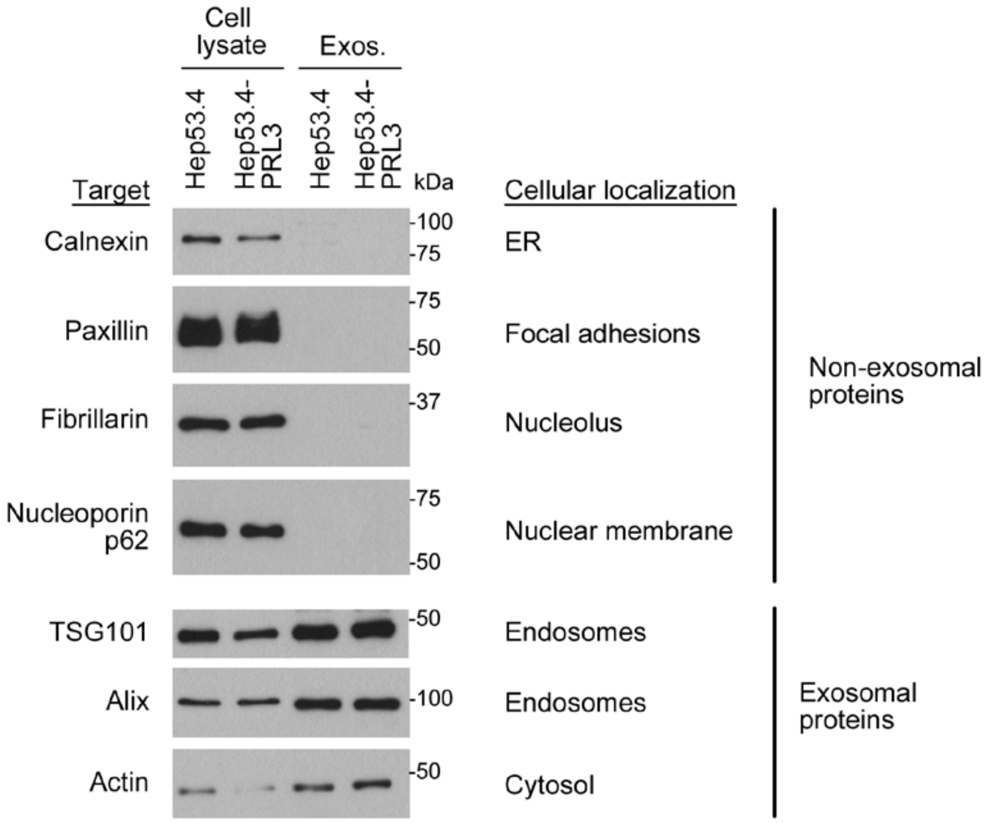

Supplementary Figure 5. Cellular proteins are selectively packaged and secreted in exosomes. Immunoblot analysis of various cellular proteins in both total cell lysates and exosomes (‘Exos.’). Source data are provided as a Source Data file.

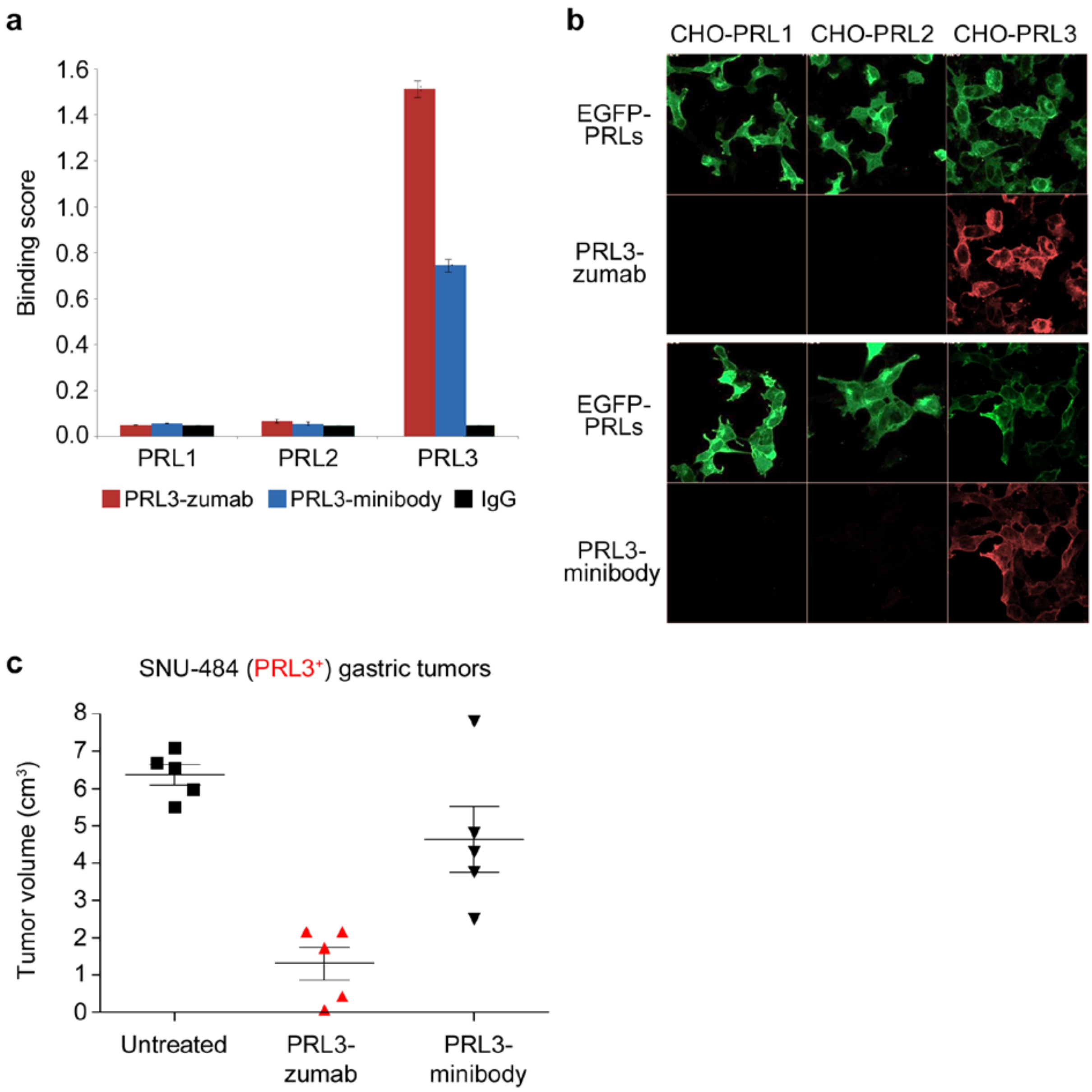

Supplementary Figure 6. PRL3-minibody retains PRL3 antigen binding specificity *in vitro* but does not significantly suppress PRL3^{+} SNU484 orthotopic gastric tumors *in vivo*. (**a**) The specific binding of PRL3-zumab, PRL3-minibody or polyclonal human IgG to purified GST-tagged human PRLs was determined by ELISA. The average binding score is derived from OD readings in triplicate wells and presented as means ± s.d.. (**b**) The specific binding of PRL3-zumab and PRL3-minibody was determined by immunofluorescence analysis of CHO cells stably expressing EGFP-tagged human PRL1 (CHO-PRL1), PRL2 (CHO-PRL2), or PRL3 (CHO-PRL3). (**c**) Mean gastric tumor volumes in untreated and treated groups of mice harboring PRL3^{+} SNU-484 orthotopic gastric tumors at Day 28. n = 5 per group; P = 0.002, one-way ANOVA; data presented as means ± s.e.m.. Source data are provided as a Source Data file.

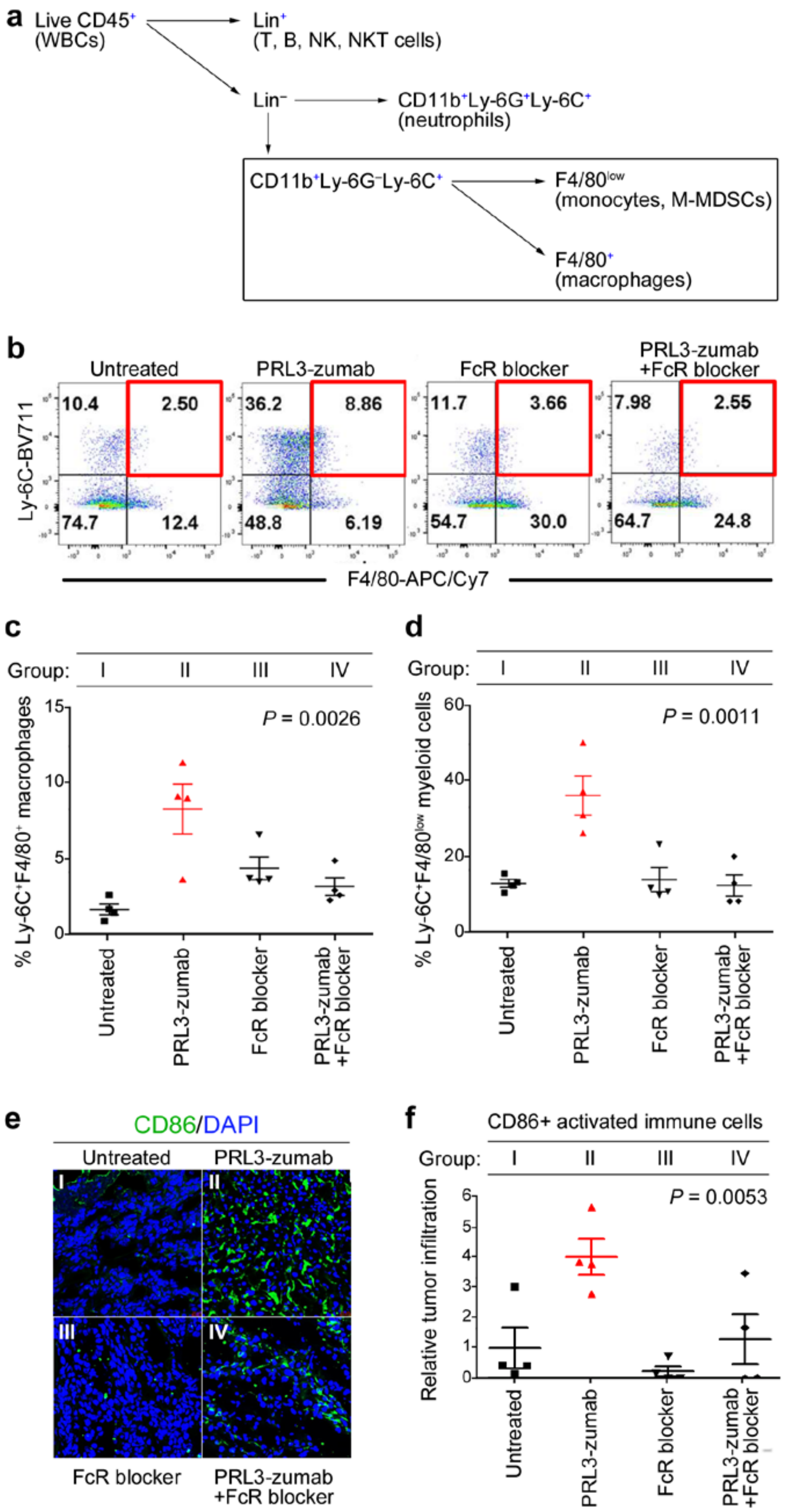

Supplementary Figure 7. PRL3-zumab promotes the accumulation of Ly-6C$^+$F4/80$^+$ macrophages, Ly-6C$^+$F4/80^{low} myeloid cells, and CD86$^+$ activated immune cells in the

tumor microenvironment in an FcR-dependent manner. (**a**) Flowchart summarizing the sequential categorization of various myeloid cell populations. (**b**) Immunoprofiling of live infiltrating $CD45^{+}Lin^{-}CD11b^{+}Ly\text{-}6G^{-}$ cells in orthotopic MHCC-LM3 liver tumor extracts from mice subjected to various treatments. $Ly\text{-}6C^{+}F4/80^{+}$ gates are highlighted with red boxes. (**c**) Summary of the mean percentage ± s.e.m. of $Ly\text{-}6C^{+}F4/80^{+}$ macrophages for each treatment group in (**b**). Data presented as means ± s.e.m., *n* = 4 independent animals per group, P-values calculated using one-way ANOVA. (**d**) Summary of the mean percentage ± s.e.m. of live $Ly\text{-}6C^{+}F4/80^{low}$ myeloid cells for each treatment group in (**b**). Data presented as means ± s.e.m., *n* = 4 independent animals per group, P-values calculated using one-way ANOVA. (**e**) Orthotopic MHCC-LM3 liver tumor tissue cryo-sections from mice subject to various treatments were analyzed by immunofluorescence with antibodies against the activated immune cell marker, CD86. Representative images are shown. Scale bar, 200 µm. (**f**) Images from (**e**) were scored for relative $CD86^{+}$ tumor infiltration. Data presented as means ± s.e.m., *n* = 4 independent animals per group, P-values calculated using one-way ANOVA. Source data are provided as a Source Data file.

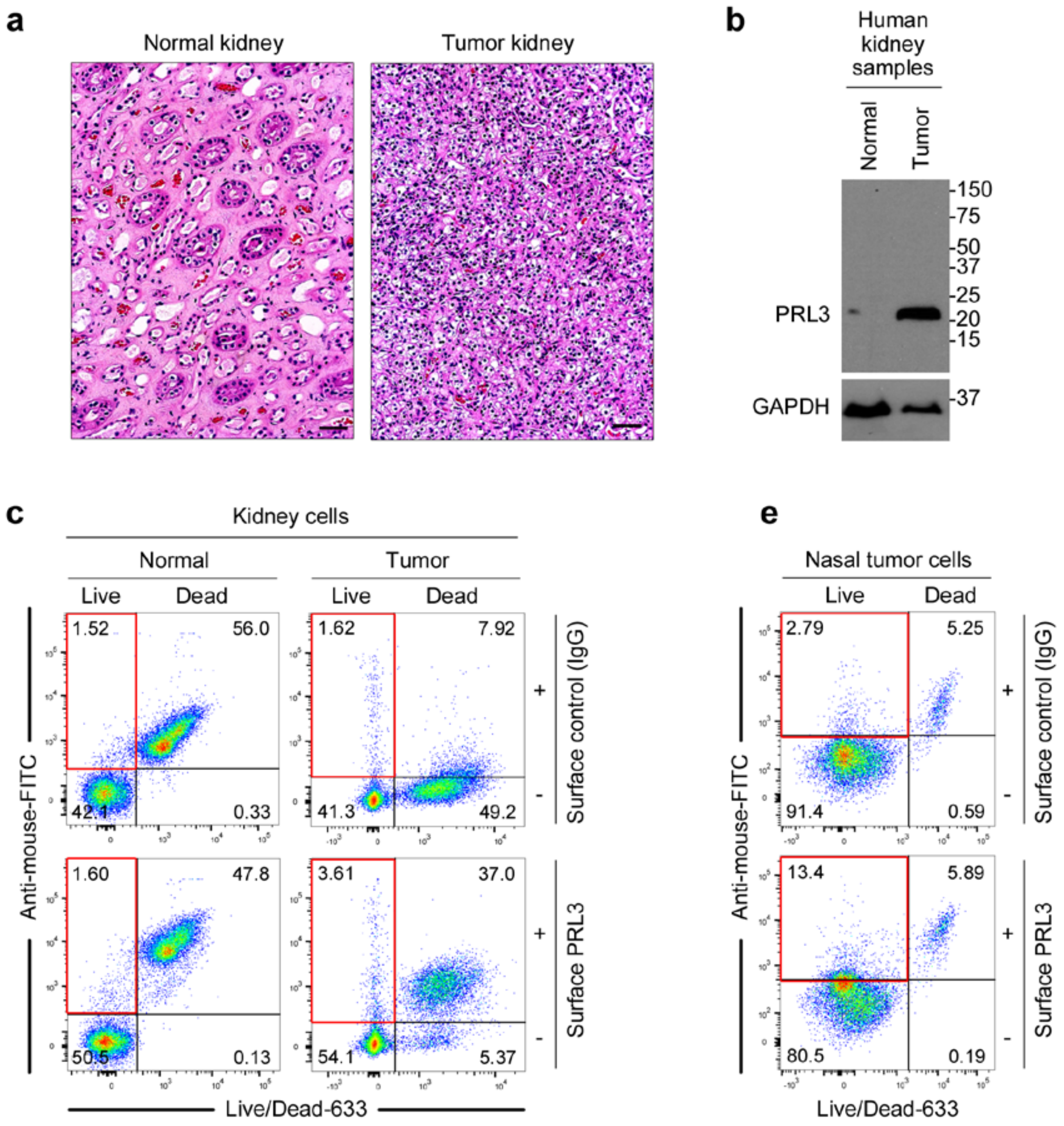

d

Sample	PRL3 WB	Surface+ (% of live cells)		
		IgG	PRL3	Adj. PRL3
Normal	Negative	3.48	3.07	0.00#
Tumor	Positive	3.77	6.26	2.48

#Values below control (hIgG) background staining reflected as zero

f

Sample	PRL-3 WB	Surface+ (% of live cells)		
		IgG	PRL3	Adj. PRL3
Nasal T.	NA	2.96	14.3	11.34

Supplementary Figure 8. PRL3 is expressed on primary human kidney and nasal tumor cells. (**a**) Hematoxylin and eosin stain of the patient-matched normal kidney sample (upper panel) and primary clear cell renal carcinoma tumor sample (lower panel). *Bar*, 400 µm. (**b**) WB for PRL3 expression. GAPDH served as a loading control. (**c**) Surface expression profiles of normal and tumor kidney cells using polyclonal mouse IgG (top panels) or mouse anti-PRL3 antibody (lower panels). (**d**) Mean percentage surface positive (surface+) live cells for each panel in (**c**) were calculated by dividing the surface antigen-positive live cells (upper left

quadrant) by total live cells (sum of both upper and lower left quadrants). The background-adjusted values of surface PRL3 (‘Adj. PRL3’) were calculated using the formula Adj. PRL3 = (%surface+ PRL3) – (%surface+ IgG) and presented in the rightmost column. For normal kidney cells, surface PRL3-specific staining was lower than negative control IgG staining and thus reported as zero. (**e**) Surface PRL3 expression profiles of nasal tumor cells using polyclonal mouse IgG (upper panel) or mouse anti-PRL3 antibody (lower panel). (**f**) Mean percentage surface positive (surface+) live cells for each panel in (**e**) were calculated by dividing the surface antigen-positive live cells by total live cells. Background-adjusted values of surface PRL3 (‘Adj. PRL3’) was calculated as in (**d**). Source data are provided as a Source Data file.

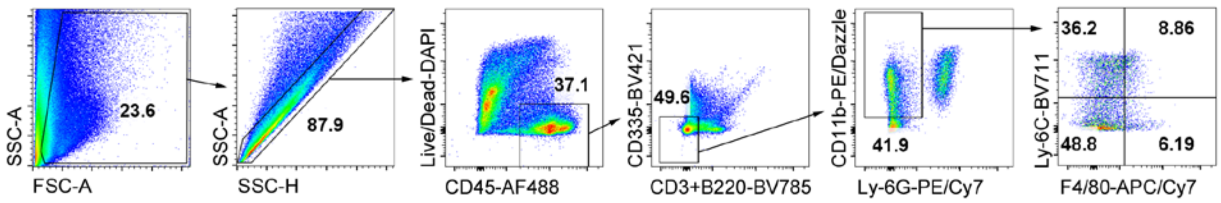

Supplementary Figure 9. Gating strategy used for identifying Ly-6C^{+}F4/80^{+} macrophages and Ly-6C^{+}F4/80^{low} myeloid cells presented in Supp. Figs. 7a-d.

SUPPLEMENTARY TABLES

Supplementary Table 1. List of publications demonstrating that PRL3 antibody therapy *in vivo* specifically inhibits $PRL3^{+}$ tumors, but not $PRL3^{-}$ tumors.

**Cell line used for tumor model**	**Cell line origin**	**PRL3 expression**	**Anti-tumor response with PRL3 antibody therapy?**	**Main text reference**
CHO-PRL-3	Ovarian	Positive	Yes	7
CT26	Colorectal	Negative	No	
B16F0	Melanoma	Positive	Yes	8
B16F10	Melanoma	Negative	No	
HCT116	Colorectal	Positive	Yes	9
A2780	Ovarian	Positive	Yes	
NCI-H460	Lung	Negative	No	
TF1-ITD	AML	Positive	Yes	10
SNU-484	Gastric	Positive	Yes	11
IM-95	Gastric	Positive	Yes	
NUGC-4	Gastric	Positive	Yes	
MKN45-PRL-3	Gastric	Positive	Yes	
MKN45	Gastric	Negative	No	

Supplementary Table 2. Details of antibodies used in this study.

Antibody/target	Species	Clone	Source	Dilution
**Western blotting**				
PRL3	Mouse	318	In-house (ref. 54)	1:2,000
GAPDH	Mouse	6C5	Millipore	1:100,000
Actin	Mouse	H-196	Santa Cruz	1:4,000
GFP	Mouse	B-2	Santa Cruz	1:4,000
TSG101	Rabbit	N/A	Proteintech	1:1,000
Alix	Mouse	3A9	Cell Signalling	1:500
Fibrillarin	Rabbit	C13C3	Cell Signalling	1:500
Calnexin	Mouse	37/Calnexin	BD Biosciences	1:500
Nucleoporin p62	Mouse	53/Nucleoporin p62	BD Biosciences	1:500
Paxillin	Mouse	165/Paxillin	BD Biosciences	1:4,000
Anti-mouse IgG, HRP-conjugated	Goat	Polyclonal	Jackson ImmunoResearch	1:4,000
Anti-human IgG, HRP-conjugated	Goat	Polyclonal	Jackson ImmunoResearch	1:4,000
Anti-rabbit IgG, HRP-conjugated	Goat	Polyclonal	Cell Signalling	1:4,000
**Immunofluorescence**				
CD335/Nkp46	Rat	29A1.4	BD Pharmingen	1:100
B220/CD45R	Rat	RA3-6B2	BD Pharmingen	1:100
CD86	Rat	GL1	BD Pharmingen	1:100
F4/80, PE-conjugated	Humanized	REA126	Miltenyi Biotec	1:50
Anti-rat IgG, Alexa Fluor 488-conjugated	Goat	Polyclonal	Invitrogen	1:200
**Flow cytometry (cell surface analysis)**				
Cetuximab (anti-EGFR)	Chimeric	C225	Merck	1:500
Polyconal human IgG	Human	Polyclonal	Bio X Cell	1:500
Polyclonal mouse IgG	Mouse	Polyclonal	Santa Cruz	1:50
Anti-mouse IgG, FITC-conjugated	Goat	Polyclonal	Jackson ImmunoResearch	1:200
Anti-human IgG, Alexa Fluor 488-conjugated	Goat	Polyclonal	Invitrogen	1:200
