# Supplementary material for: PRL3-zumab as an immunotherapy to inhibit tumors expressing PRL3 oncoprotein
Source: Nat Commun. 2019 Jun 6;10:2484. doi: 10.1038/s41467-019-10127-x (PMC6554295; doi:10.1038/s41467-019-10127-x)
Supplement: Supplementary file 3 — Reporting Summary [file 41467_2019_10127_MOESM3_ESM.pdf]

nature research

Corresponding author(s): Qi Zeng

Last updated by author(s): Apr 22, 2019

Reporting Summary

Nature Research wishes to improve the reproducibility of the work that we publish. This form provides structure for consistency and transparency in reporting. For further information on Nature Research policies, see Authors & Referees and the Editorial Policy Checklist.

Statistics

For all statistical analyses, confirm that the following items are present in the figure legend, table legend, main text, or Methods section.

n/a	Confirmed	
☐	☒	The exact sample size (*n*) for each experimental group/condition, given as a discrete number and unit of measurement
☐	☒	A statement on whether measurements were taken from distinct samples or whether the same sample was measured repeatedly
☐	☒	The statistical test(s) used AND whether they are one- or two-sided<br>*Only common tests should be described solely by name; describe more complex techniques in the Methods section.*
☒	☐	A description of all covariates tested
☒	☐	A description of any assumptions or corrections, such as tests of normality and adjustment for multiple comparisons
☐	☒	A full description of the statistical parameters including central tendency (e.g. means) or other basic estimates (e.g. regression coefficient) AND variation (e.g. standard deviation) or associated estimates of uncertainty (e.g. confidence intervals)
☒	☐	For null hypothesis testing, the test statistic (e.g. *F*, *t*, *r*) with confidence intervals, effect sizes, degrees of freedom and *P* value noted<br>*Give P values as exact values whenever suitable.*
☒	☐	For Bayesian analysis, information on the choice of priors and Markov chain Monte Carlo settings
☒	☐	For hierarchical and complex designs, identification of the appropriate level for tests and full reporting of outcomes
☒	☐	Estimates of effect sizes (e.g. Cohen's *d*, Pearson's *r*), indicating how they were calculated

Our web collection on statistics for biologists contains articles on many of the points above.

Software and code

Policy information about availability of computer code

Data collection	*Provide a description of all commercial, open source and custom code used to collect the data in this study, specifying the version used OR state that no software was used.*
Data analysis	*Provide a description of all commercial, open source and custom code used to analyse the data in this study, specifying the version used OR state that no software was used.*

For manuscripts utilizing custom algorithms or software that are central to the research but not yet described in published literature, software must be made available to editors/reviewers. We strongly encourage code deposition in a community repository (e.g. GitHub). See the Nature Research guidelines for submitting code & software for further information.

Data

Policy information about availability of data

All manuscripts must include a data availability statement. This statement should provide the following information, where applicable:

- Accession codes, unique identifiers, or web links for publicly available datasets
- A list of figures that have associated raw data
- A description of any restrictions on data availability

All data generated or analysed during this study are included in this published article (and its supplementary information files). The source data underlying all Main and Supplementary Figures are provided as a Source Data file

Field-specific reporting

Please select the one below that is the best fit for your research. If you are not sure, read the appropriate sections before making your selection.

☒ Life sciences ☐ Behavioural & social sciences ☐ Ecological, evolutionary & environmental sciences

For a reference copy of the document with all sections, see nature.com/documents/nr-reporting-summary-flat.pdf

Life sciences study design

All studies must disclose on these points even when the disclosure is negative.

Sample size	For human tissue samples, we analyzed all samples that were provided by our collaborators (based on availability).<br>For animal group sizes, we used power analysis to determine the minimum number of animals required for significance based on the following calculation parameters: 1) difference in mean >= 50% (estimated according to previous experiments), 2) p value < 0.05, and 3) power = 80%.
Data exclusions	No data were excluded from analyses.
Replication	Our attempts to replicate experiments were successful.
Randomization	Tumor-inoculated mice were randomly allocated into various treatment groups. Human tissue samples were randomly provided for analysis based on availability.
Blinding	For some human tissue samples, we were blinded as to whether the material provided by our collaborators were from tumor or normal areas, and received this information only after analysis.

Reporting for specific materials, systems and methods

We require information from authors about some types of materials, experimental systems and methods used in many studies. Here, indicate whether each material, system or method listed is relevant to your study. If you are not sure if a list item applies to your research, read the appropriate section before selecting a response.

Materials & experimental systems

n/a	Involved in the study	
☐	☒	Antibodies
☐	☒	Eukaryotic cell lines
☒	☐	Palaeontology
☐	☒	Animals and other organisms
☒	☐	Human research participants
☒	☐	Clinical data

Methods

n/a	Involved in the study	
☒	☐	ChIP-seq
☐	☒	Flow cytometry
☒	☐	MRI-based neuroimaging

Antibodies

Antibodies used

1) Murine anti-PRL-3 mAb, clone 318, generated in-house
2) GAPDH, Millipore, CB1001, clone 6C5, Lot 2987549
3) Actin, Santa Cruz , sc 47778, clone H-196, Lot K1716
4) GFP, Santa Cruz, sc 9996, clone B-2, Lot K1808
5) TSG101, Proteintech, 14497-1-AP, clone Ag5920, Lot 00057871
6) Alix, Cell Signalling, 2171, clone 3A9, Lot 2
7) Fibrillarin, Cell Signalling, 432, clone C13C3, Lot 1
8) Calnexin, BD Biosciences, 610523, clone 37/Calnexin (RUO), Lot 0000070374-B
9) Nucleoporin p62, BD Biosciences, 610497, clone 53/Nucleoporin p62, Lot 23815
10) Paxillin, BD Biosciences, 610051, clone 165 /Paxillin, Lot 23816
11) Anti-mouse IgG HRP conjugated, JacksonImmunoResearch, 315035046 ,polyclonal, Lot 129408
12) Anti-human IgG HRP conjugated, JacksonImmunoResearch,709035098 , polyclonal, Lot 135103
13) Anti-rabbit IgG HRP conjugated, Cell Signalling, 7074P2, polyclonal, Lot 26
14) CD335/ Nkp46 , BD Pharmingen, 560757, clone 29A1.4, Lot 4330688
15) B220/CD45R, BD Pharmingen, 553090, Clone RA3-6B2 (RUO), Lot 3032645
16) CD86, BD Pharmingen , 553691, clone GL1, Lot 4308834
17) F4/80 PE, MACS, 130-116-499, Clone REA126, Lot 5181121216
18) anti-rat IgG Alexa Fluor 488 conjugated, Invitrogen, A11006, polyclonal, Lot 1728142
19) Cetuximab (anti-EGFR), Merck, DB00002 (BTD00071, BIOD00071), clone C225, Lot 135620
20) Polyclonal human IgG, Bio X cell, BE0092, polyclonal, Lot 81314TGDM/0315
21) Polyclonal mouse IgG, Santa Cruz, sc-2025, polyclonal, Lot#H-1512

	22) Anti-mouse IgG FITC-conjugated, Jackson ImmunoResearch, 115-095-003, polyclonal, Lot 116324<br>23) Anti-human IgG Alexa Fluor 488 conjugated, Invitrogen, A11013, polyclonal, Lot 1946343<br>24) PRL3-zumab, generated by Wuxi Biologics
Validation	1) Murine anti-PRL-3 mAb clone 318; validation reference: Li et al, Clin. Cancer Res. 11:2195-2204 (2005)<br>2) GAPDH, Millipore, CB1001; Validation based on manufacturer's data sheet<br>3) Actin, Santa Cruz , sc 47778, Validation based on manufacturer's data sheet<br>4) GFP, Santa Cruz, sc 9996, Validation based on manufacturer's data sheet<br>5) TSG101, Proteintech, 14497-1-AP, Validation based on manufacturer's data sheet<br>6) Alix, Cell Signalling, 2171, Validation based on manufacturer's data sheet<br>7) Fibrillarin, Cell Signalling, Validation based on manufacturer's data sheet<br>8) Calnexin, BD Biosciences, 610523, Validation based on manufacturer's data sheet<br>9) Nucleoporin p62, BD Biosciences, 610497, Validation based on manufacturer's data sheet<br>10) Paxillin, BD Biosciences, 610051, Validation based on manufacturer's data sheet<br>14) CD335/ Nkp46 , BD Pharmingen, 560757, Validation based on manufacturer's data sheet<br>15) B220/CD45R, BD Pharmingen, 553090, Validation based on manufacturer's data sheet<br>16) CD86, BD Pharmingen , 553691, Validation based on manufacturer's data sheet<br>17) F4/80 PE, MACS, 130-116-499, Validation based on manufacturer's data sheet<br>19) Cetuximab (anti-EGFR), Merck, DB00002 (BTD00071, BIOD00071), Validation based on manufacturer's data sheet<br>24) PRL3-zumab, validation reference: Thura et al, JCI Insight 1:e87607 (2016)

Eukaryotic cell lines

Policy information about cell lines

Cell line source(s)	Liver cancer cell lines MHCC-LM3 (National Cancer Center Singapore, NCCS)<br>Hep3B2.1 (American Type Culture Collection (ATCC), HB-8064)<br>HepG2 (ATCC, HB-8065)<br>PLC (ATCC, CRL-8024)<br>Huh-7 (Japanese Collection of Research Bioresources, JCRB)<br>SNU449 (#00449,Korea Cell Line Bank, KCLB)<br>Hep53.4 (Cell Line Service, CLS, 400200)<br>SNU-484 gastric cancer cell line (KCLB)<br>B16F0 mouse melanoma cell line (ATCC, CRL6322)<br>CHO-K1 cell line (ATCC, CCL61)
Authentication	None of the cell lines used were authenticated.
Mycoplasma contamination	All the cell lines were tested negative for mycoplasma contamination using a PCR-based mycoplasma test kit.
Commonly misidentified lines<br>(See ICLAC register)	None of the cell lines used are listed in the ICLAC Register (version 9).

Animals and other organisms

Policy information about studies involving animals; ARRIVE guidelines recommended for reporting animal research

Laboratory animals	NcR nude mice, males, 7-9 weeks old
Wild animals	The study did not involve wild animals.
Field-collected samples	The study did not involve the samples collected from the field.
Ethics oversight	Animal studies were approved by the A*STAR Institutional Animal Care and Use Committee (IACUC; Study No.: 161130) and performed in accordance with approved guidelines and regulations.

Note that full information on the approval of the study protocol must also be provided in the manuscript.

Flow Cytometry

Plots

Confirm that:

☒ The axis labels state the marker and fluorochrome used (e.g. CD4-FITC).

☒ The axis scales are clearly visible. Include numbers along axes only for bottom left plot of group (a 'group' is an analysis of identical markers).

☒ All plots are contour plots with outliers or pseudocolor plots.

☒ A numerical value for number of cells or percentage (with statistics) is provided.

Methodology

Sample preparation	Freshly-harvested orthotopic MHCC-LM3 liver tumors and human tissues samples were dissociated using a human Tumor Dissociation Kit and gentle MACS Octo Dissociator (Miltenyi Biotec) and filtered with 70µm cell strainers (Fisher Scientific #22363548) to obtain single cell suspensions. Dissociated cells were washed once with PBS, counted, resuspended in RPMI, and kept on ice till analysis.<br><br>For analysis of cultured cells in vitro, exponentially-growing cells at 80% confluence in T-75 flasks were washed once with PBS and incubated with 2 mL non-enzymatic cell dissociation buffer (Sigma-Aldrich) for 5 minutes to dislodge the adherent cells into suspension. In serum-starvation experiments, cells were washed twice with PBS and incubated with serum-free RPMI at 37°C and 5% CO2 for the indicated durations prior to harvest. Harvested cells were washed once with PBS, counted, resuspended in RPMI, and kept on ice till analysis.
Instrument	BD LSR II
Software	FlowJo 10.5.3
Cell population abundance	NA. No cells were sorted.
Gating strategy	Supplementary figure 9 provides the sequential gating strategy for identification of Ly-6C+F4/80+ macrophages and Ly-6C +F4/80- myeloid cells.

☒ Tick this box to confirm that a figure exemplifying the gating strategy is provided in the Supplementary Information.
